# Quantifying the Adhesion of Silicate Glass–Ceramic Coatings onto Alumina for Biomedical Applications

**DOI:** 10.3390/ma12111754

**Published:** 2019-05-30

**Authors:** Francesco Baino

**Affiliations:** 1 Institute of Materials Physics and Engineering, Applied Science and Technology Department, Politecnico di Torino, Corso Duca degli Abruzzi 24, 10129 Torino, Italy; francesco.baino@polito.it; Tel.: +39-011-090-4668; 2 Interuniversity Center for the promotion of the 3Rs Principles in Teaching and Research, Italy

**Keywords:** biomaterials, bioceramics, coating, mechanical properties

## Abstract

Deposition of bioactive glass or ceramic coatings on the outer surface of joint prostheses is a valuable strategy to improve the osteointegration of implants and is typically produced using biocompatible but non-bioactive materials. Quantifying the coating–implant adhesion in terms of bonding strength and toughness is still a challenge to biomaterials scientists. In this work, wollastonite (CaSiO_3_)-containing glass–ceramic coatings were manufactured on alumina tiles by sinter-crystallization of SiO_2_–CaO–Na_2_O–Al_2_O_3_ glass powder, and it was observed that the bonding strength decreased from 34 to 10 MPa as the coating thickness increased from 50 to 300 µm. From the viewpoint of bonding strength, the coatings with thickness below 250 µm were considered suitable for biomedical applications according to current international standards. A mechanical model based on quantized fracture mechanics allowed estimating the fracture toughness of the coating on the basis of the experimental data from tensile tests. The critical strain energy release rate was also found to decrease from 1.86 to 0.10 J/m^2^ with the increase of coating thickness, which therefore plays a key role in determining the mechanical properties of the materials.

## 1. Introduction

Deposition of glass or ceramic coatings on orthopedic and dental implants is useful to impart better biocompatibility and bone-bonding properties [1] to the underlying material, which typically exhibits high mechanical performance but is not inherently bioactive (e.g., titanium and its alloys [2,3]). Furthermore, the coatings have a protective function, reducing implant corrosion (especially in the case of metallic implants) and protecting living tissues against corrosion products [4].

Plasma-sprayed hydroxyapatite coatings have been used for many years on titanium implants in orthopedic and dental surgery [5]; however, the rise of some concerns about the stability of the calcium phosphate layer (interfacial delamination) has stimulated the search for new options, like bioactive glasses. These materials have the unique capability of forming a tight bond with bone and soft collagenous tissues through a series of chemical reactions conceptually similar to the mechanism of conventional glass corrosion [6]. Hence, bioactive glasses are, by nature, reactive in aqueous solution and are prone to degrade over time according to a wide range of dissolution kinetics that strongly depend on the glass composition. The risk of uncontrolled dissolution, carrying the problem of prosthesis mobility in the long term [7], is perhaps the major reason why the application of bioactive glass coatings on permanent implants is still very limited in clinics and, to date, an ideal bioactive material for coating does not exist, which motivates further research.

The literature on biomedical glass coatings is mainly focused on both the search for effective deposition methods, which evolved from conventional enameling [8] to electrophoretic deposition [9] and laser-based methods [10], and the evaluation of coating degradation, biocompatibility, and osteointegration [11]. The mechanical performance of the coating, being quite difficult to quantitatively assess, has often been neglected. The crack path resulting from interfacial indentation was studied in order to evaluate coating resistance; however, this approach does not allow the adhesion strength to be quantitatively measured, thereby making unreliable the comparison between different systems [12,13]. Fracture toughness has also been estimated by measuring the crack length in indentation tests; however, these methods may be prone to significant errors due to violations of model assumptions during practical testing situations, especially when brittle coatings are tested [14,15]. Overall, the existing literature witnesses that quantifying the coating adhesion and toughness is still a challenge but should be crucial in the development of new glass coatings.

In this work, it is studied how thickness can affect the mechanical performance of silicate glass–ceramic coatings deposited onto alumina. To the best of the author’s knowledge, it is the first time that such a combination of coating material (SiO_2_–CaO–Na_2_O–Al_2_O_3_ base glass) and substrate (alumina) is quantitatively studied from this viewpoint. Specifically, the bonding strength of the coatings was experimentally assessed by performing tensile tests, and the fracture toughness was then determined by applying a properly derived model based on quantized fracture mechanics.

## 2. Materials and Methods

### 2.1. Glass Preparation

The starting silicate glass (57SiO_2_–34CaO–6Na_2_O–3Al_2_O_3_ wt.%), previously developed for biomedical applications [16], was produced by a melting-quenching route. Firstly, the reagents (analytical-grade powders of SiO_2_, CaCO_3_, Na_2_CO_3_, and Al_2_O_3_, all purchased from Sigma-Aldrich, St. Louis, MO, USA) were homogenously mixed for 30 min in an orbital shaker; then, the blend of powders was transferred to a platinum crucible, heated to 1150 °C (heating rate 10 °C/min) in an electrically heated furnace, and melted for 1 h in air. The melt was quenched into deionized water to obtain a frit that was ball-milled (Pulverisette 0, Fritsch, Idar-Oberstein, Germany) and sieved by a stainless-steel sieve (Giuliani Technology Srl, Turin, Italy) to a final particle size below 32 μm.

### 2.2. Coating Production

Glass coatings of different nominal thicknesses (50, 100, 150, 200, 250, and 300 µm) were manufactured on flat high-purity alumina tiles (size 10 mm × 10 mm, thickness 1 mm; produced by diamond-cutting of 200 mm × 200 mm AL603103 99% Al_2_O_3_ sheets, Goodfellow, Coraopolis, PA, USA) by gravity-guided deposition after suspending proper amounts of glass powder in ethanol. The glass particles were left to deposit on the substrates overnight; then, the containers hosting the materials were placed in an oven at 90 °C for 6 h to remove the excess ethanol and allow complete dying of the “greens”. The samples were then thermally treated in an electrically heated furnace at 1000 °C for 3 h (heating rate 5 °C/min) in order to allow glass particle sintering and coating consolidation.

### 2.3. Characterizations

Differential thermal analysis (DTA) was performed on the glass powder to determine the characteristic temperatures of the material, i.e., glass transition temperature (T_g_), crystallization onset (T_x_), crystallization peak (T_p_), and melting (T_m_). Specifically, glass powder was analyzed by using a DTA 404 PC instrument (Netzsch, Selb, Germany) in the temperature range of 25 to 1400 °C with a heating rate of 10 °C/min. After being introduced into a platinum crucible provided by the instrument manufacturer, 50 mg of glass underwent the thermal cycle; an equal amount of high-purity Al_2_O_3_ powder was put in the reference crucible. Standard calibration procedure and baseline corrections were performed.

Sintered coatings underwent X-ray diffraction (XRD) in the 2θ-range of 10 to 60° by using a X’Pert Pro PW3040/60 diffractometer (PANalytical, Eindhoven, The Netherlands) operating at 40 kV, 30 mA with Cu Kα incident radiation (wavelength 0.15405 nm), step size 0.02°, and counting time 1 s per step. Crystalline phase assignment was performed by using X’Pert HighScore software 2.2b (PANalytical, Eindhoven, The Netherlands) equipped with the PCPDFWIN database (http://pcpdfwin.updatestar.com).

The coating cross sections and the substrate–coating interface were also inspected by scanning electron microscopy (SEM). For this purpose, the samples were embedded in epoxy resin (Epofix, Struers, Ballerup, Denmark), cut by a diamond wheel (Accutom, Struers, Ballerup, Denmark), and polished using #600 to #4000 SiC grit paper; the resulting cross sections were sputter-coated with silver and analyzed by field-emission SEM (Supra^TM^ 40, Zeiss, Oberkochen, Germany) at an accelerating voltage of 15 kV. The thickness of the coating was measured directly on the SEM images by making use of an image-analysis software provided by the manufacturer; the results were expressed as average ± standard deviation of three measurements per each sample. Compositional analysis was performed by energy dispersive spectroscopy (EDS); the probe was included in the SEM equipment.

The tensile (bonding) strength was measured by applying tensile loads to pull the coating apart, following the relevant ASTM standard [17]. Before being tested using an MTS machine (cross-head speed 1 mm/min), each sample was glued to two stainless-steel cylindrical fixtures (diameter 16 mm) by a strong bi-component adhesive (Araldite^®^ AV 119, Huntsman, Woodlands, TX, USA), according to an experimental procedure described elsewhere [16]. The bonding strength (*σ*) was calculated as the maximum load (*F*) per unit cross-sectional area (*A*):

(1) $$\sigma =\frac{F}{A}$$

Pull-out tests were performed on five specimens per each of the six sample batches having different nominal thickness; the results were expressed as average ± standard deviation.

## 3. Toughness Estimation: Derivation of the Mechanical Model

An estimation of the coating toughness was obtained from the values of tensile strength (see Equation (1)) by implementing an appropriate model, based on fracture mechanics, which was derived as follows. The theory of linear elastic fracture mechanics states that the total potential energy of a system can be expressed as:

(2) Π=U−W

where *U* is the strain energy, and *W* is the work done by the external force *F*. Considering our case, *U* and *W* can be calculated as:

(3) $$U=\frac{1}{2}F^{2}\left(\frac{1}{k_{1}}+\frac{1}{k_{2}}\right)$$

(4) $$W=F^{2}\left(\frac{1}{k_{1}}+\frac{1}{k_{2}}\right)$$

where *k*_1_ and *k*_2_ are the stiffness of alumina tile and coating, respectively, before crack propagation.

Hence, after combining Equations (2)–(4), it is obtained:

(5) $$\Pi =-\frac{1}{2}F^{2}\left(\frac{1}{k_{1}}+\frac{1}{k_{2}}\right)$$

Griffith’s energy criterion states that crack propagation occurs when the variation of the total potential energy dΠ, corresponding to a virtual increment of crack surface dA, is equal to the energy needed to create the new free crack surface, i.e., $d\Pi =-G_{IC}dA$, where *G_IC_* is the fracture energy (per unit area created) of the material.

The recent theory of quantized fracture mechanics extends the Griffith’s criterion to discrete cracks: thus, after substituting differentials with finite differences (i.e., d→Δ), the Griffith’s equation can be rewritten as [18]:

(6) $$G_{IC}=-\frac{\Delta \Pi }{\Delta A}$$

There are three possible failure modes occurring when the coatings undergo pull-out tests: in fact, the fracture can be (i) totally adhesive, (ii) totally cohesive, or (iii) mixed.

Considering the case (i), it is ideally assumed that the failure occurs at the coating–alumina interface; therefore, the variation of the total potential energy is:

(7) $$\Delta \Pi =-\frac{1}{2}F^{2}\left[\left(\frac{1}{k_{1}^{*}}-\frac{1}{k_{1}}\right)+\left(\frac{1}{k_{2}^{*}}-\frac{1}{k_{2}}\right)\right]$$

where $k_{1}^{*}$ and $k_{2}^{*}$ are the stiffness after crack propagation.

The compliances in Equation (7) can be expressed as $\frac{1}{k_{1}^{*}}-\frac{1}{k_{1}}=\frac{t_{1}}{E_{1}A^{2}}\cdot \frac{\Delta A}{1-\left(\frac{\Delta A}{A}\right)}$ and $\frac{1}{k_{2}^{*}}-\frac{1}{k_{2}}=\frac{t_{2}}{E_{2}A^{2}}\cdot \frac{\Delta A}{1-\left(\frac{\Delta A}{A}\right)}$, where *E*_1_ (=400 GPa) and *E*_2_ (=90 GPa [19]) are the Young’s moduli of alumina and coating, respectively, and *t*_1_ (=1 mm) and *t*_2_ the corresponding thicknesses.

Thus, Equations (7) can be rewritten as:

(8) $$\Delta \Pi =-\frac{1}{2}F^{2}\left(\frac{t_{1}}{E_{1}A^{2}}\cdot \frac{\Delta A}{1-\left(\frac{\Delta A}{A}\right)}+\frac{t_{2}}{E_{2}A^{2}}\cdot \frac{\Delta A}{1-\left(\frac{\Delta A}{A}\right)}\right)$$

Combining Equations (6) and (8), the energy release rate *G_I_*_,12_ can be expressed as:

(9) $$G_{I,12}={\left(\sigma_{I,12}\right)}^{2}\frac{E_{1}t_{2}+E_{2}t_{1}}{2E_{1}E_{2}\left(1-\left(\frac{\Delta A}{A}\right)\right)}$$

Hence, the delamination (critical) strength can be expressed as:

(10) $$\sigma_{IC,12}=\sqrt{\frac{2E_{1}E_{2}}{E_{1}t_{2}+E_{2}t_{1}}G_{IC,12}\left(1-\frac{\Delta A}{A}\right)}$$

Considering the case (ii), it is ideally assumed that the failure occurs entirely in the coating; this means that the tensile strength of the coating material ($\sigma_{IC,2}$ = 47 MPa [19]) is lower than the adhesion strength at the coating–substrate interface.

A simple visual inspection of the fracture surfaces after tensile tests revealed that there was a coexistence of the two failure modes (i) and (ii) (adhesive + cohesive, resulting in mode (iii)). Therefore, the critical stress is assumed to be predicted by a mean field approach as:

(11) $$\sigma_{IC}=\sigma =\sigma_{IC,12}\frac{A_{d}}{A}+\sigma_{IC,2}\left(1-\frac{A_{d}}{A}\right)$$

where $A_{d}=A-\Delta A$ is the delamination area.

After combining Equations (10) and (11), it is finally obtained:

(12) $$G_{IC,12}=\frac{{\left[\sigma -\sigma_{IC,2}\left(1-\frac{A_{d}}{A}\right)\right]}^{2}}{\frac{2E_{1}E_{2}}{E_{1}t_{2}+E_{2}t_{1}}{\left(\frac{A_{d}}{A}\right)}^{3}}$$

## 4. Results and Discussion

The silicate glass used in this work was initially proposed for potential usage in the field of bone regeneration and substitution [16]. This composition was then discarded with respect to such applications, as the high content of SiO_2_ and the significant presence of Al_2_O_3_ resulted in an almost inert behavior with almost no apatite-forming capability in contact with biological fluids, thereby preventing the formation of a tight bond to bone. However, the material was found highly suitable to produce porous orbital implants [20], which must elicit minimal or no reactions in contact with ocular tissues [21], and multilayer coatings onto ceramic implants [22].

**Figure 1:**
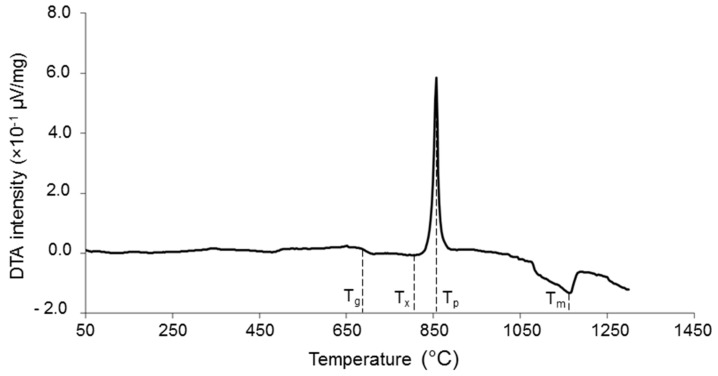
DTA plot of the starting glass powder used to manufacture the coatings.

The characteristic temperatures of the glass, determined from the DTA plot (Figure 1), were T_g_ = 685 °C (inflection point), T_x_ = 800 °C (crystallization onset), T_p_ = 855 °C (exothermic peak), and T_m_ = 1155 °C (endothermic peak).

**Figure 2:**
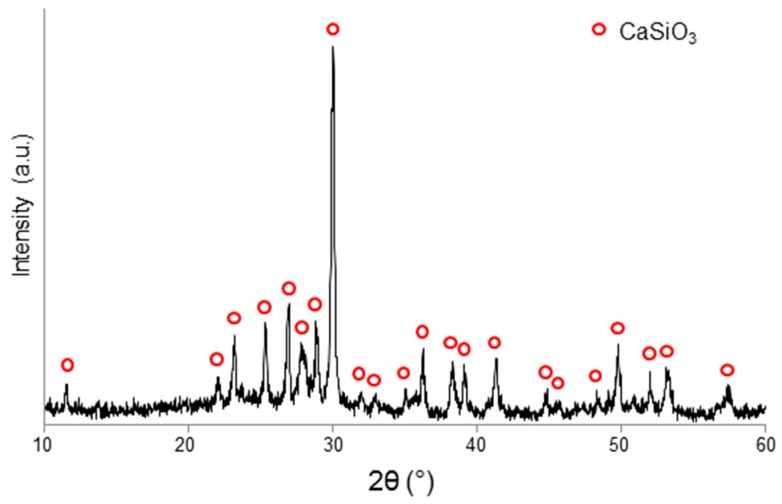
XRD pattern of a sintered coating (1000 °C/3 h).

The coatings underwent devitrification upon thermal treatment, as shown in Figure 2. The development of wollastonite crystals (CaSiO_3_, PDF code no. 00-027-0088) when the material was thermally treated at 1000 °C is consistent with the results from thermal analysis, showing that crystallization started at 855 °C. The XRD results were also in agreement with those obtained on the same glass system after heat treatment above 900 °C [23]. No problems of possible cytotoxicity due to wollastonite were forecast, as this crystalline phase was clearly proved to be highly biocompatible and suitable in contact with living bone, also for filling load-bearing osseous defects [24].

**Figure 3:**
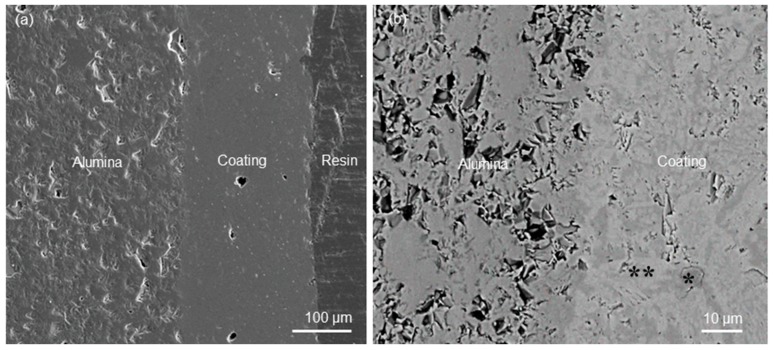
Analysis of the coating cross section: (**a**) SEM micrograph in “secondary” mode (400×) and (**b**) high-magnification detail (2500×) inspected in back-scattering mode showing the interface between coating and alumina as well as the coating microstructure (glass–ceramic nature); the white areas (**) correspond to the wollastonite crystals, while the dark zones (*) correspond to the residual glassy phase.

Analysis of the cross section revealed that the interface between coating and alumina tile was flawless without any apparent interfacial crack or delamination in intact samples (Figure 3a). However, some isolated small pores could be observed in the coating. The quality of adhesion between coating and alumina tile after thermal treatment was very good due to an almost perfect matching between the thermal expansion coefficients of glass (8.7 × 10^−6^ °C^−1^ [16]) and alumina (8.5 × 10^−6^ °C^−1^).

The thicknesses of the coatings, reported in Table 1, were in good agreement with the nominal values. The slight decrease compared to the nominal thickness can be attributed to the glass powder densification that occurred during sintering.

The SEM micrograph shown in Figure 3b, acquired in back-scattering mode, clearly shows the glass–ceramic nature of the coating, in which wollastonite crystals and residual glassy phase coexisted. This was further confirmed by the compositional analyses (EDS): in fact, the CaSiO_3_ crystals (“white” areas with atomic composition 43.2% Si, 56.8% Ca), exhibiting a typical acicular shape, were embedded in a grey matrix in which high amounts of Al and Na could be found, too, along with low Ca content (atomic composition 22.6% Na, 13.1% Al, 58.9% Si, 5.4% Ca), corresponding to the residual glass. These results are in accordance with the XRD pattern (Figure 2), where a bump in the 2θ-range of 20 to 35° can be observed, which is the typical proof of the presence of an amorphous phase. The back-scattering SEM image was analyzed by the free software ImageJ version 2016 (https://imagej.nih.gov/ij/index.html) to obtain a rough quantification of crystalline and glassy phase proportions in the glass–ceramic material: the white regions corresponding to CaSiO_3_ crystals were estimated to be about 70% of the total cross-sectional area of the coating.

**Figure 4:**
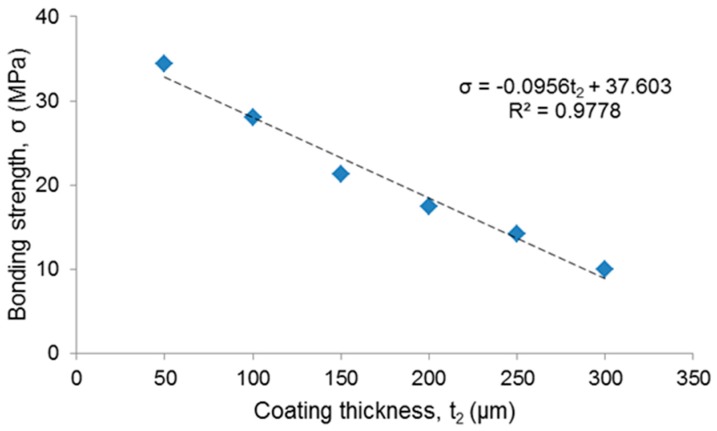
Relationship between bonding strength and coating thickness (blue points: experimental values, dashed line: fitting curve).

The results from the tensile tests showed that the bonding strength decreased as the coating thickness increased (Table 1). Data interpolation by the least-squares method suggested a linear relationship between bonding strength and thickness of the coating (Figure 4); the high value of the coefficient R^2^ also confirmed the good fitting of the interpolating function with the experimental data.

No specific international standard exists about the minimum bonding strength recommended for biomedical glass coatings; however, a tensile strength of 15 MPa is prescribed by ISO 13779 for hydroxyapatite coatings on surgical implants [25]. This requirement was fulfilled by the coatings produced in this work with thickness below 250 µm (Table 1).

**Figure 5:**
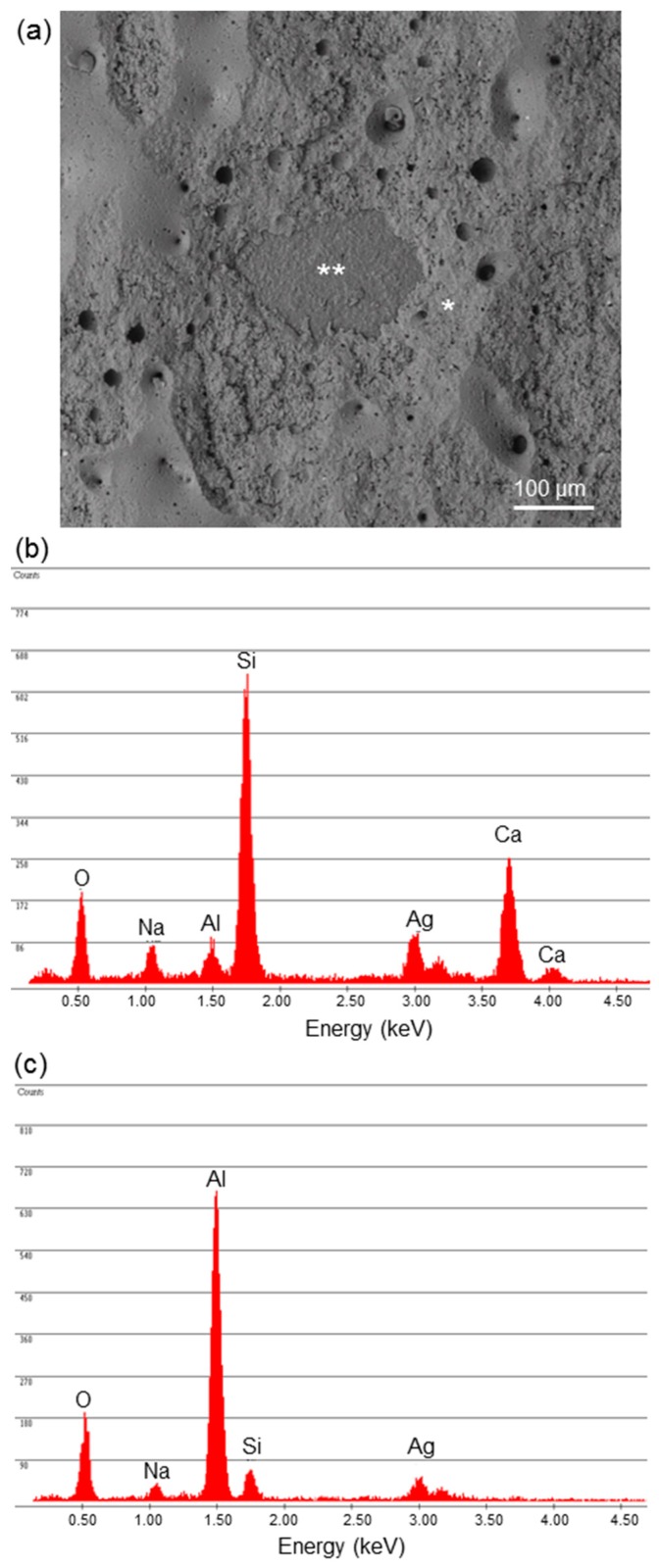
Analysis of fracture surface: (**a**) SEM micrograph showing the morphology of the coating after tensile test and (**b**,**c**) compositional assessment to identify the areas of adhesive and cohesive fracture. The region marked with (*) in (**a**) corresponds to the fracture area inside the coating (the corresponding EDS pattern in (**b**) reveals the presence of all the typical elements of the glass composition, i.e., Si, Ca, Na, and Al), while the region marked with (**) corresponds to interfacial delamination (a high peak for Al is visible in the corresponding EDS pattern in (**c**), with just small traces of Si and Na).

Analysis of the fracture surfaces (Figure 5) confirmed a mixed failure mode (adhesive + cohesive failure), consistently with the assumption behind Equation (11). The critical strain energy release rate assessed by applying Equation (12) decreased as the coating thickness increased (Table 1). Since the *G_IC_*_,12_ value can be interpreted as an estimation of fracture toughness [18], this means that the thicker coatings were less tough than the thin ones. This trend is consistent with previous observations reported in the literature about glass and ceramic coatings deposited onto metallic implants, albeit a clear quantification of the trend has been seldom reported. Furthermore, to the best of the author’s knowledge, no study has been specifically conducted to determine the toughness–thickness relationship in glass coatings deposited on bioceramic substrates, which was attempted for the first time in the present work.

Gomez-Vega et al. [12,26] claimed that thinner silicate bioactive glass and composite coatings on titanium implants were significantly less prone to interfacial cracking but did not provide any quantitative assessment of bonding strength and fracture toughness. Zhao et al. [10] produced bioactive SiO_2_–CaO–Na_2_O–P_2_O_5_–MgO glass coatings by pulsed laser deposition on Ti6Al4V alloy and observed that thick coatings showed poorer adhesion to the substrate. Matinmanesh et al. [27] reported a similar trend for SiO_2_–CaO–Na_2_O–P_2_O_5_–ZnO coatings on Ti6Al4V alloy, too, and demonstrated that increments in the coating thickness led to higher residual stresses that tended to increase the “crack driving force”. This interesting explanation can be extended to the present work. The residual stresses in the coating contributed to the release of the stored strain energy during the tensile test and, therefore, less energy from the applied loads was needed to reach the critical value required for crack propagation. In other words, lower tensile loads were required to overcome the resistance of thicker coatings against cracking because of higher residual stresses.

Wollastonite-containing glass–ceramic coatings (thickness 140 µm) deposited on alumina by airbrushing in a previous work exhibited a fracture toughness of 0.8 J/m^2^ [23], which is very close to the value reported in Table 1 for the 150 µm-thick samples.

**Figure 6:**
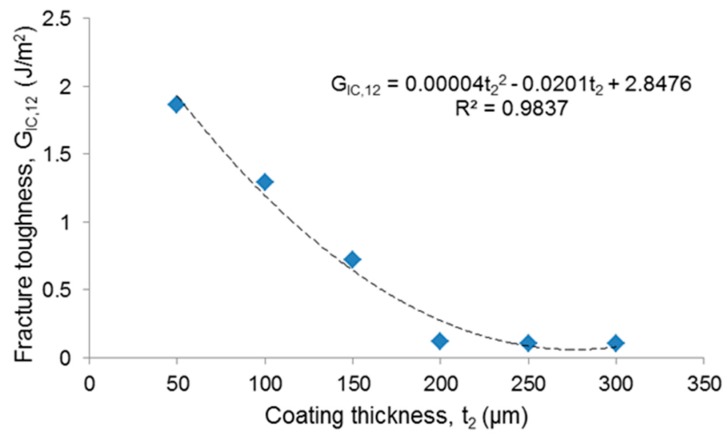
Relationship between fracture toughness and coating thickness (blue points: experimental values, dashed line: fitting curve).

Data interpolation by the least-squares method suggested a quadratic (polynomial) relationship between fracture toughness and thickness of the coating (Figure 6), as confirmed by the high value of the coefficient R^2^. A significant drop of *G_IC_*_,12_ values accompanied by a decrease of *A_d_* (Table 1) could be observed if the coating thickness exceeded 150 µm, which could be considered as a threshold value to take into account at the design stage of the coating.

## 5. Conclusions

The bonding strength and critical strain energy release rate of wollastonite-containing glass–ceramic coatings were found to decrease as the coating thickness increased, i.e., thinner coatings proved to have higher resistance against fracture than thicker ones. These relationships were quantified by combining experimental data from tensile tests on the coatings and a model based on quantized fracture mechanics. The obtained functions are very valuable to link the coating thickness, which can be tailored at the design and manufacturing stages, with those key mechanical characteristics. Specifically, it could be predicted what is the maximum thickness allowed so that the coating can exhibit the mechanical characteristics required for a given application. Therefore, the coating thickness should be carefully taken into account in order to optimize the material performance and design. The results also suggest that, ideally, the coating should be as thin as possible for increasing the mechanical performance; thus, further improvements are forecast if the coating thickness decreases below the minimum value (50 µm) considered in this study. The same methodological approach followed to estimate toughness could also be applied to different glass or ceramic coatings based on other compositions of biomedical interest.

## Figures and Tables

**Table 1:** Mechanical properties of the coatings.

Nominal/Measured Thickness of the Coating (µm)	*A_d_* (mm^2^)	*σ* (MPa)	*G_IC_*_,12_ (J/m^2^)
50/48 ± 5	92.0 ± 4.3	34.4 ± 3.1	1.86 ± 0.35
100/94 ± 6	85.0 ± 3.7	28.0 ± 1.6	1.29 ± 0.19
150/142 ± 8	86.3 ± 6.0	21.3 ± 2.6	0.72 ± 0.29
200/194 ± 10	72.0 ± 2.5	17.4 ± 1.1	0.12 ± 0.063
250/240 ± 8	71.2 ± 8.4	14.2 ± 3.2	0.11 ± 0.013
300/296 ± 8	71.4 ± 3.6	10.0 ± 1.2	0.10 ± 0.076

## References

[1] McEntire B.J., Bal B.S., Rahaman M.N., Chevalier J., Pezzotti G. (2015). Ceramics and ceramic coatings in orthopaedics. J. Eur. Ceram. Soc..

[2] Chen X., Li H.S., Yin Y., Feng Y., Tan X.W. (2015). Macrophage proinflammatory response to the Ti alloy equipment in dental implantation. Genet. Mol. Res..

[3] Gibon E., Amanatullah D.F., Loi F., Pajarinen J., Nabeshima A., Yao Z., Hamadouche M., Goodman S.B. (2017). The biological response to orthopaedic implants for joint replacement: Part I: Metals. J. Biomed. Mater. Res. B.

[4] Sola A., Bellucci D., Cannillo V., Cattini A. (2011). Bioactive glass coatings: A review. Surf. Eng..

[5] Sun L., Berndt C.C., Gross K.A., Kucuk A. (2001). Material fundamentals and clinical performance of plasma-sprayed hydroxyapatite coatings: A review. J. Biomed. Mater. Res. (Appl. Biomater.).

[6] Fernandes H.R., Gaddam A., Rebelo A., Brazete D., Stan G.E., Ferreira J.M.F. (2018). Bioactive glasses and glass-ceramics for healthcare applications in bone regeneration and tissue engineering. Materials.

[7] Alonso-Barrio J.A., Sanchez-Herraez S., Fernandez-Hernandez O., Betegon-Nicolas J., Gonzalez-Fernandez J.J., Lopez-Sastre A. (2004). Bioglass-coated femoral stem. J. Bone Jt. Surg..

[8] Peddi L., Brow R.K., Brown R.F. (2008). Bioactive borate glass coatings for titanium alloys. J. Mater. Sci. Mater. Med..

[9] Boccaccini A.R., Keim S., Ma R., Li Y., Zhitomirsky I. (2010). Electrophoretic deposition of biomaterials. J. R. Soc. Interface.

[10] Zhao Y., Song M., Liu J. (2008). Characteristics of bioactive glass coatings obtained by pulsed laser deposition. Surf. Interface Anal..

[11] Baino F., Verné E. (2017). Glass-based coatings on biomedical implants: A state-of-the-art review. Biomed. Glasses.

[12] Gomez-Vega J.M., Saiz E., Tomsia A.P., Marshall G.W., Marshall S.J. (2000). Bioactive glass coatings with hydroxyapatite and bioglass particles on Ti-based implants. 1. Processing. Biomaterials.

[13] Lopez-Esteban S., Saiz E., Fujino S., Oku T., Suganuma K., Tomsia A.P. (2003). Bioactive glass coatings for orthopedic metallic implants. J. Eur. Ceram. Soc..

[14] Kruzic J.J., Kim D.K., Koester K.J., Ritchie R.O. (2009). Indentation techniques for evaluating the fracture toughness of biomaterials and hard tissues. J. Mech. Behav. Biomed. Mater..

[15] Hsiung C.H.H., Pyzik A.J., Gulsoy E.B., De Carlo F., Xiao X., Faber K.T. (2013). Impact of doping on the mechanical properties of acicular mullite. J. Eur. Ceram. Soc..

[16] Vitale-Brovarone C., Baino F., Tallia F., Gervasio C., Verné E. (2012). Bioactive glass-derived trabecular coating: A smart solution for enhancing osteointegration of prosthetic elements. J. Mater. Sci. Mater. Med..

[17] ASTM C633 (2017). Standard Test Method for Adhesion or Cohesion Strength of Thermal Spray Coatings. https://www.astm.org/Standards/C633.htm.

[18] Pugno N., Ruoff R. (2004). Quantized fracture mechanics. Philos. Mag..

[19] Chen Q., Baino F., Pugno N.M., Vitale-Brovarone C. (2013). Bonding strength of glass-ceramic trabecular-like coatings to ceramic substrates for prosthetic applications. Mater. Sci. Eng. C.

[20] Baino F. (2018). Porous glass-ceramic orbital implants: A feasibility study. Mater. Lett..

[21] Baino F. (2015). How can bioactive glasses be useful in ocular surgery?. J. Biomed. Mater. Res. A.

[22] Baino F., Vitale-Brovarone C. (2015). Trabecular coating on curved alumina substrates using a novel bioactive and strong glass-ceramic. Biomed. Glasses.

[23] Baino F., Vitale-Brovarone C. (2015). Wollastonite-containing bioceramic coatings on alumina substrates: Design considerations and mechanical modelling. Ceram. Int..

[24] Kokubo T., Ito S., Sakka S., Yamamuro T. (1986). Formation of a high strength bioactive glass-ceramic in the system MgO–CaO–SiO2–P_2_O_5_. J. Mater. Sci..

[25] ISO 13779-4 (2018). Implants for Surgery: Hydroxyapatite, Part 4: Determination of Coating Adhesion Strength. https://www.iso.org/standard/64619.html.

[26] Gomez-Vega J.M., Saiz E., Tomsia A.P. (1999). Glass-based coatings for titanium implant alloys. J. Biomed. Mater. Res..

[27] Matinmanesh A., Rodriguez O., Towler M.R., Zalzal P., Schemitsch E.H., Papini M. (2016). Quantitative evaluation of the adhesion of bioactive glasses onto Ti6Al4V substrates. Mater. Des..

